# Integrated pharmacovigilance assessment of drug-associated cholangitis: signal detection, clinical characterization, and mechanistic insights

**DOI:** 10.3389/fphar.2026.1891926

**Published:** 2026-07-30

**Authors:** Maohe Yu, Hao Wang, Lve Cheng, Xinlang Wu, Qujin Li, Junwei Niu, Haowen Li, Shengwei Li

**Affiliations:** Department of Hepatobiliary Surgery, The Second Affiliated Hospital of Chongqing Medical University, Chongqing, China

**Keywords:** adverse drug reactions, biliary disease, cholangitis, disproportionality analysis, drug safety, FAERS, pharmacovigilance

## Abstract

**Background:**

Drug-related cholangitis has been increasingly recognized in clinical practice; however, the spectrum of implicated agents and their relative contributions remain incompletely characterized.

**Objective:**

To systematically identify drugs associated with cholangitis reporting and to characterize disproportionality signals and time-to-onset patterns using a large pharmacovigilance database.

**Methods:**

Data were extracted from the FDA Adverse Event Reporting System (FAERS) from the first quarter of 2004 to the second quarter of 2025. Disproportionality analysis was performed using reporting odds ratios (RORs) with 95% confidence intervals (CIs). Significant signals were defined as ROR >1, with the lower bound of the 95% CI > 1, and a Bonferroni correction was applied for multiple comparisons. A case/non-case design was used for regression analyses. Candidate drugs were selected using the least absolute shrinkage and selection operator (LASSO) regression and further evaluated in a multivariable logistic regression model. Time-to-onset was assessed using descriptive statistics and visualized using distribution plots.

**Results:**

A total of 17,083 reports of drug-related cholangitis were identified. Eighty-four drugs showed significant disproportionality signals, predominantly involving antineoplastic agents, immunomodulators, and antidiabetic drugs. Multivariable analysis identified older age, female sex, and 21 drugs as factors associated with increased reporting odds of cholangitis reporting within the FAERS database. These findings should be interpreted as pharmacovigilance signals intended for hypothesis generation rather than confirmation of causal relationships. The median time to onset was 97 days (interquartile range [IQR], 7–102 days), with most cases occurring within the first 3 months after treatment initiation.

**Conclusion:**

This large-scale pharmacovigilance study identified multiple drug classes associated with increased reporting odds of cholangitis and demonstrated an early-onset pattern. These findings highlight the importance of clinical vigilance during the initial treatment period and provide a basis for future mechanistic and epidemiological studies.

## 1 Introduction

Cholangitis encompasses a spectrum of biliary disorders ranging from acute infectious inflammation (An et al., 2021; Miura et al., 2018; Sulzer and Ocuin, 2019; Wada et al., 2007) to chronic fibroinflammatory conditions such as sclerosing cholangitis (Khoshpouri et al., 2019; Ludwig et al., 2023). Despite advances in diagnostic and therapeutic strategies, cholangitis remains associated with substantial morbidity and mortality, particularly when diagnosis or intervention is delayed (Brooling and Leal, 2017; Kirstein et al., 2020; Miura et al., 2018; Visentin et al., 2018).

Medications have increasingly been recognized as potential contributors to biliary injury (Ludwig et al., 2023). Drug-related cholangitis may arise through multiple mechanisms, including immune-mediated injury (Kusakabe et al., 2019), direct toxicity to biliary epithelial cells, microvascular impairment (Barnett and Malafa, 2001), and alterations in bile composition or flow (Colvin et al., 2022; Cozma et al., 2024). Several drug classes, such as immune checkpoint inhibitors, tyrosine kinase inhibitors (Björnsson and Andrade, 2022; Pi et al., 2021), antibiotics, and hormonal agents (Cirillo et al., 2005; Ml et al., 1998), have been implicated in biliary adverse events. However, existing evidence is largely derived from case reports and small observational studies, limiting the ability to comprehensively characterize the spectrum of implicated drugs.

Large-scale pharmacovigilance databases provide an opportunity to systematically evaluate rare but clinically important adverse events. The FDA Adverse Event Reporting System (FAERS) is a widely used global database for signal detection and hypothesis generation in drug safety research (Rodriguez et al., 2001).

In this study, we conducted a comprehensive pharmacovigilance analysis using FAERS to identify drugs associated with reports of cholangitis. We further characterized disproportionality signals, evaluated time-to-onset patterns, and explored potential mechanistic pathways. These findings aim to enhance clinical awareness and inform future research on drug-related biliary injury.

## 2 Materials and methods

### 2.1 Data source and study design

This retrospective pharmacovigilance study was conducted using data from the U.S. Food and Drug Administration (FDA) Adverse Event Reporting System (FAERS), covering the period from the first quarter of 2004 to the second quarter of 2025. FAERS is a spontaneous reporting database designed to support post-marketing safety surveillance and signal detection for adverse drug reactions.

The database consists of seven core datasets, including patient demographic information (DEMO), adverse events coded using the Medical Dictionary for Regulatory Activities (MedDRA) terminology (REAC), drug information (DRUG), outcomes (OUTC), report sources (RPSR), therapy dates (THER), and indications (INDI).

This study followed a retrospective case/non-case pharmacovigilance design and was conducted in accordance with current recommendations for disproportionality analyses, including the READUS-PV reporting framework. Because FAERS contains publicly available anonymized data, institutional review board approval and informed consent were not required.

### 2.2 Case identification and data cleaning

Duplicate reports were identified and removed according to FDA-recommended procedures. When multiple reports shared the same CASEID, the report with the most recent FDA_DT was retained. If both CASEID and FDA_DT were identical, the report with the higher PRIMARYID was selected. Additional manual deduplication procedures were applied to reduce residual duplicate records.

Cases were defined as FAERS reports containing cholangitis-related MedDRA preferred terms (PTs), including “cholangitis,” “acute cholangitis,” “chronic cholangitis,” “sclerosing cholangitis,” “immune-mediated cholangitis,” “infective cholangitis,” and related PTs. Only reports in which the suspected drug was classified as a primary suspect (PS) drug were included to improve specificity.

Non-cases were defined as all remaining reports in the FAERS database that did not contain cholangitis-related PTs.

To minimize indication bias and disease-related confounding, drugs primarily indicated for the treatment of cholangitis, biliary tract infection, or hepatobiliary obstruction were excluded from signal detection analyses when appropriate. Reports lacking essential identifiers or containing implausible chronological information were excluded from time-to-onset analyses.

Because FAERS is a spontaneous reporting database, missing demographic and clinical information was not imputed. Missing values were retained for descriptive analyses but excluded from regression analyses when necessary.

### 2.3 Signal detection and disproportionality analysis

Disproportionality analyses were performed using reporting odds ratios (RORs) with corresponding 95% confidence intervals (CIs) to identify drug-event reporting imbalances within the FAERS database.

A standard two-by-two contingency table (Supplementary Table S1) approach was used to compare the proportion of cholangitis reports associated with a given drug against the proportion observed for all other drugs in the database. A disproportionality signal was considered statistically significant when the ROR was >1, and the lower limit of the 95% CI exceeded 1.

To reduce false-positive findings arising from large-scale multiple comparisons, the Bonferroni correction was applied to p-values generated using Fisher’s exact test. In addition, a more stringent threshold (ROR ≥3 combined with Bonferroni-adjusted p < 0.01) was used to identify stronger and potentially more clinically relevant pharmacovigilance signals. The ROR was calculated as follows:

$$ROR=\frac{a/c}{b/d}\;ROR_{95\%CI}=e^{\ln\left(ROR\right)\pm 1.96\sqrt{\left(\frac{1}{a}+\frac{1}{b}+\frac{1}{c}+\frac{1}{d}\right)}}$$

Because disproportionality analyses may be influenced by reporting trends, indication bias, channeling bias, and stimulated reporting, the resulting signals were interpreted as hypothesis-generating observations rather than evidence of causal relationships.

### 2.4 Variable selection and regression analysis

Drugs were considered eligible for regression analyses if they met the predefined signal detection criteria, including a lower limit of the 95% confidence interval (CI) of the reporting odds ratio (ROR) greater than 1, a Bonferroni-adjusted p value < 0.01, and at least 100 cholangitis reports. These criteria were applied to reduce instability arising from sparse data and to prioritize pharmacovigilance signals with greater statistical robustness.

In addition, an ROR threshold greater than three was selected to identify stronger pharmacovigilance signals. Higher disproportionality values are generally considered to indicate more robust reporting associations and may improve signal specificity by reducing weaker or spurious associations. This threshold was applied for signal prioritization and descriptive purposes only and was not intended to infer causality.

Eligible drugs were subsequently entered into a least absolute shrinkage and selection operator (LASSO) regression model with 10-fold cross-validation for variable selection. Variables retained by LASSO, together with demographic covariates (age and sex), were included in a multivariable logistic regression model. Older age was predefined as age ≥42 years according to commonly used geriatric epidemiological criteria.

The dependent variable was defined as the presence or absence of cholangitis reports (case versus non-case). Adjusted odds ratios (ORs) and corresponding 95% confidence intervals (CIs) were calculated. Given the observational nature of FAERS data, the regression analyses were intended to identify factors associated with increased reporting odds of cholangitis rather than establish causal relationships.

### 2.5 Time-to-onset analysis

Time-to-onset (TTO) was defined as the interval between the recorded therapy start date and the onset date of cholangitis.

Only reports containing complete and chronologically plausible therapy initiation and event onset dates were included in TTO analyses. Records with missing dates, invalid intervals, or implausible chronological sequences were excluded.

TTO was summarized using descriptive statistics, including medians and interquartile ranges (IQRs), and visualized using distribution plots and cumulative incidence curves to characterize temporal reporting patterns following drug initiation.

### 2.6 Sensitivity analyses

Several sensitivity analyses were performed to assess the robustness and consistency of the observed disproportionality signals.

First, analyses were repeated after restricting reports to those submitted by healthcare professionals to reduce potential consumer reporting bias. Second, analyses were repeated using only reports with complete demographic information. Third, drugs primarily used in hepatobiliary malignancies or advanced gastrointestinal cancers were selectively excluded in exploratory analyses to further evaluate potential indication-related confounding.

Finally, signal consistency was evaluated under more stringent disproportionality thresholds (ROR ≥3 and Bonferroni-adjusted p < 0.01). These analyses were intended to evaluate signal stability rather than to confirm causal associations.

### 2.7 Bias mitigation and methodological considerations

Several methodological considerations were incorporated to improve the interpretability of pharmacovigilance signals.

To reduce indication bias, drugs directly indicated for cholangitis or biliary disorders were excluded from primary signal detection analyses where appropriate. Restriction to primary suspect (PS) drugs was used to improve specificity and reduce confounding from concomitant therapies.

Potential notoriety bias and stimulated reporting were also considered, particularly for immune checkpoint inhibitors and recently marketed therapies that have received increased clinical attention regarding hepatobiliary adverse events. Because FAERS lacks detailed denominator data and comprehensive clinical information, residual confounding by underlying disease severity, malignancy status, biliary interventions, or concomitant medications could not be fully excluded.

Accordingly, all identified signals were interpreted cautiously as pharmacovigilance observations requiring further validation through epidemiological and mechanistic studies.

### 2.8 Statistical analysis

Continuous variables were summarized using medians and interquartile ranges (IQRs), whereas categorical variables were expressed as counts and percentages.

All statistical analyses were conducted using R software (version 4.5.2). Statistical significance was defined as a two-sided p-value <0.05 unless otherwise specified. Bonferroni-adjusted p-values were used in disproportionality analyses to reduce the risk of false-positive findings resulting from multiple testing.

## 3 Results

### 3.1 Baseline characteristics of drug-related cholangitis

**FIGURE 1:**
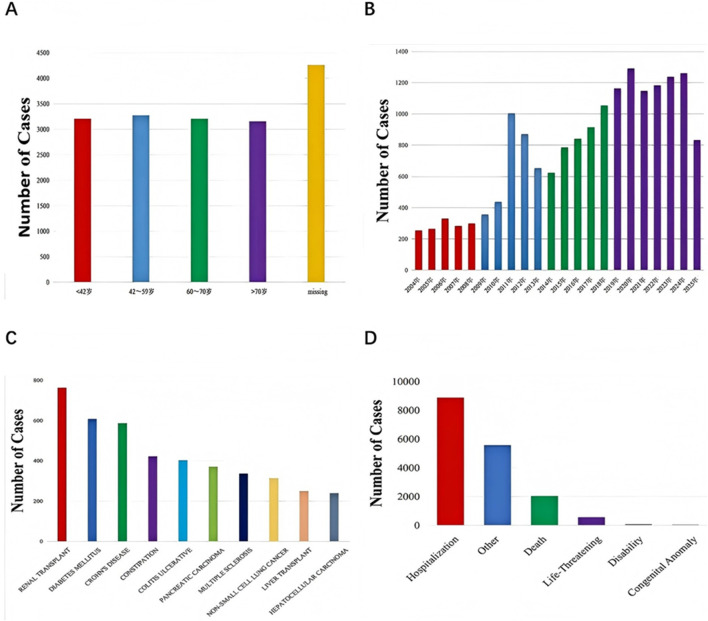
Relevant information from various drug-related cholangitis reports. **(A)** Drug-related cholangitis reports by patient age group; **(B)** Reporting rate of drug-related cholangitis by year; **(C)** Diagnosis of patients with drug-related cholangitis; **(D)** Clinical outcomes with drug-related cholangitis.

During the study period, a total of 23,168,942 adverse event reports were retrieved from the FAERS database. After deduplication and application of the predefined inclusion and exclusion criteria, 19,149,650 unique reports were retained for further analysis. Among these, 17,083 reports involving cholangitis were identified and included in the final analysis set. The baseline characteristics are summarized in Table 1; Figure 1.

**TABLE 1:** Basic patient information.

Characteristics	Drug-related Cholangitis (N = 17,083)
AGE	
Median (Q1, Q3)	59.0 (42.0, 70.0)
Unknown	4256 (24.9%)
Gender	
Female	8451 (49.5%)
Male	7111 (41.6%)
Unknown	1521 (8.9%)
Weight	
Median (Q1, Q3)	70.75 (58.96, 86.0)
Unknown	10,659 (62.4%)
Occupation of the reporter	
Physician	6920 (40.5%)
Consumer	4635 (27.1%)
Health professional	1474 (8.6%)
Pharmacist	738 (4.3%)
Unknown	3316 (19.4%)
Country of the reporter	
United States	5894 (34.5%)
Japan	3112 (18.2%)
Canada	1230 (7.2%)
Germany	1017 (6.0%)
France	954 (5.6%)
United Kingdom	645 (3.8%)
Country not specified	481 (2.8%)
Italy	294 (1.7%)
Spain	224 (1.3%)
Australia	162 (0.9%)
Netherlands	149 (0.9%)
China	149 (0.9%)
Brazil	141 (0.8%)
Austria	125 (0.7%)
Colombia	120 (0.7%)
Other	2387 (14.0%)

Unknown indicates a missing value. Missing values were retained for descriptive analyses but excluded from regression analyses.

Among these reports, 8,451 (49.5%) involved female patients and 7,111 (41.6%) involved male patients, while sex information was missing in 1,521 (8.9%) cases. The median age was 59 years (interquartile range [IQR], 42–70), and the median body weight was 70.75 kg (IQR, 58.96–86.0). Missing data were present for age (24.9%) and weight (62.4%).

Physicians were the most frequent reporters (6,920, 40.5%), followed by consumers (4,635, 27.1%), other health professionals (1,474, 8.6%), and pharmacists (738, 4.3%). Reports were most commonly submitted from the United States, Japan, and several European countries.

The annual number of reports showed an increasing trend over time, with a peak observed in 2020 (Figure 1B). By age group, reports were most frequent among patients aged 42–59 years (Figure 1A). Kidney transplant recipients accounted for the highest number of drug-related cholangitis cases (Figure 1C). Regarding outcomes, hospitalization was reported in 8,869 cases (51.9%), death in 2,030 cases (11.9%), and other serious outcomes in 5,567 cases (32.6%) (Figure 1D).

The temporal increase in cholangitis reports observed around 2020 should be interpreted cautiously. Changes in reporting behavior, increased awareness of drug-induced hepatobiliary injury, publication of emerging safety signals, and growing utilization of immune checkpoint inhibitors and targeted therapies may have contributed to reporting frequency. Therefore, annual report counts should not be interpreted as direct measures of disease incidence.

### 3.2 Drugs associated with cholangitis

**FIGURE 2:**
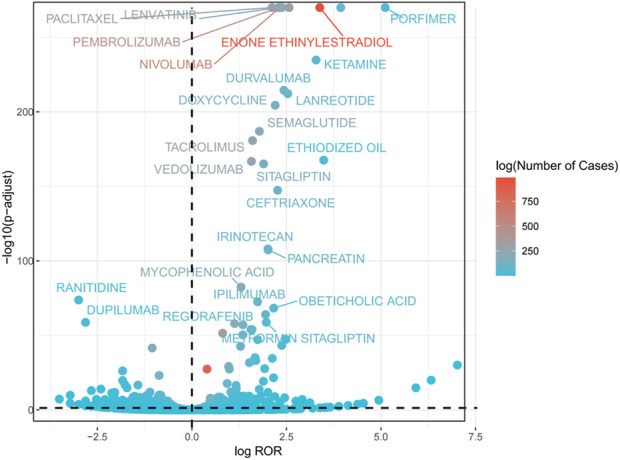
Volcano plot of drugs associated with cholangitis. ROR, reporting odds ratio; P-adjust, p-value after Bonferroni correction.

A volcano plot illustrating the association between suspected drugs and cholangitis reporting is presented in Figure 2. The x-axis represents the logarithm of the reporting odds ratio (log[ROR]), and the y-axis represents the negative logarithm of the Bonferroni-adjusted p-value (−log10 [P-adjust]). Each point corresponds to an individual drug, and the color gradient reflects the number of reported cases.

A total of 84 drugs met the predefined criteria for significant disproportionality signals (Supplementary Table S2). To further characterize the strength of disproportionality signals, drugs were ranked according to their reporting odds ratios (RORs). The five strongest signals identified in the present analysis were porfimer (ROR = 166.04), ursodeoxycholic acid (ROR = 51.17), ethiodized oil (ROR = 32.64), drospirenone/ethinylestradiol (ROR = 29.41), and ketamine (ROR = 26.68), indicating markedly elevated reporting disproportionality for cholangitis compared with other detected agents.

These top-ranked signals represented diverse pharmacological categories, including photosensitizing agents, bile acid preparations, iodinated contrast media, hormonal therapies, and anesthetic agents, suggesting heterogeneous and potentially distinct mechanisms underlying drug-associated cholangitis.

These drugs were distributed across multiple therapeutic classes, including antineoplastic agents, immunomodulators, antidiabetic drugs, and anti-infective agents.

### 3.3 Disproportionality signals and regression analysis

**FIGURE 3:**
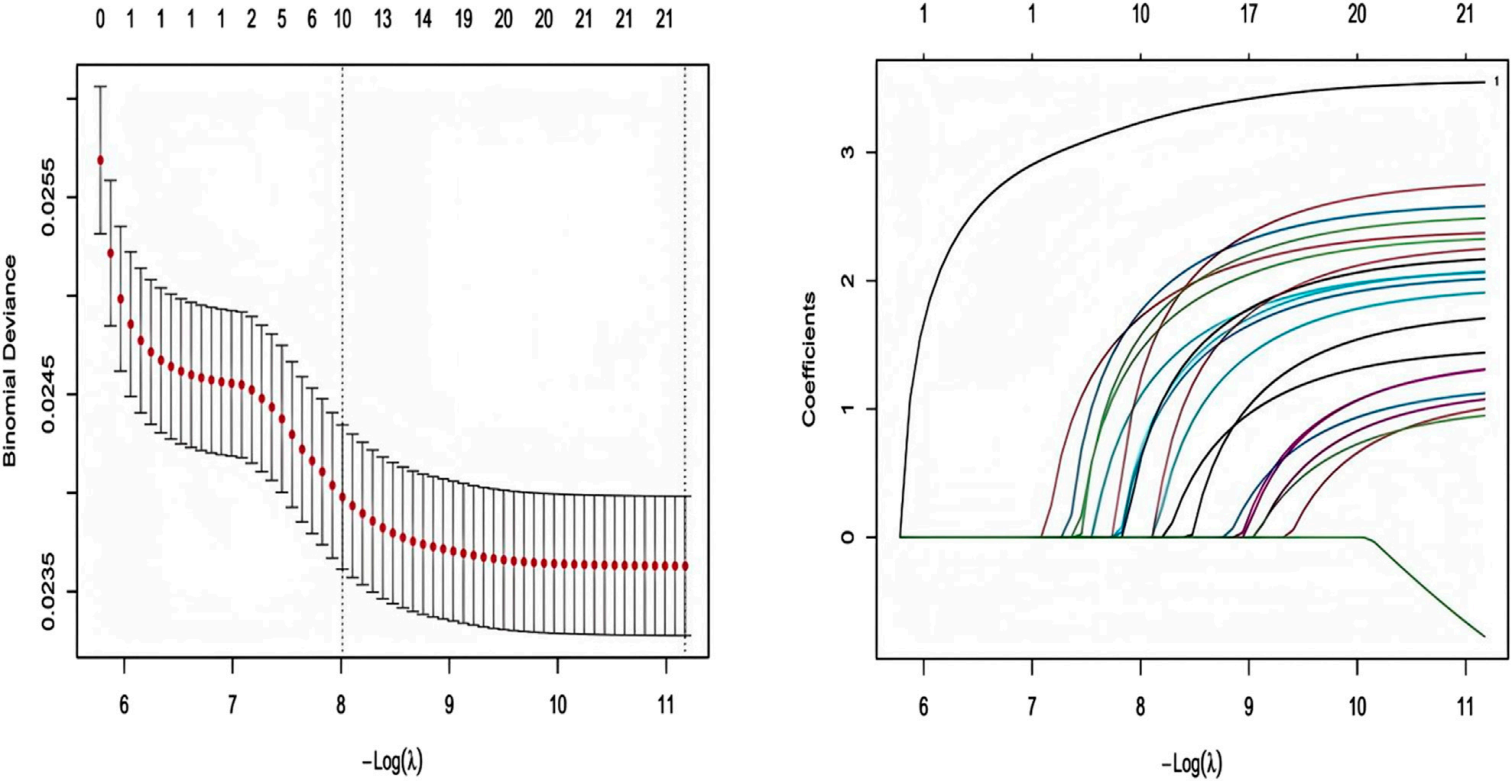
Variable selection using LASSO regression. LASSO, least absolute shrinkage and selection operator.

Drugs meeting the predefined thresholds (number of reports >100, lower limit of the 95% CI of the ROR >1, and adjusted p-value <0.01) were included in the univariable analysis. Variables satisfying these criteria were subsequently entered into LASSO regression for variable selection (Figure 3).

**FIGURE 4:**
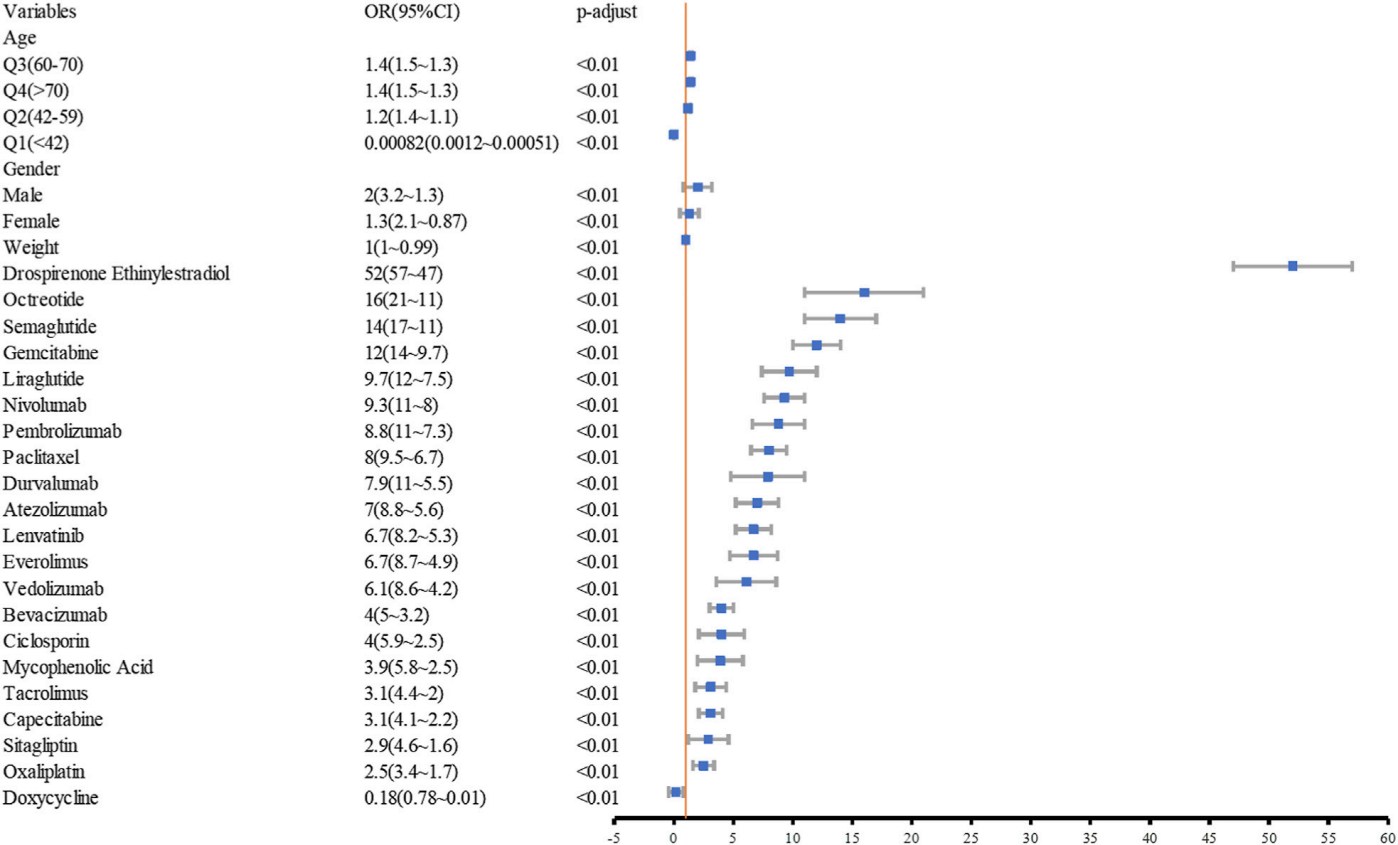
Multivariable logistic regression analysis of disproportionality signals. CI, confidence interval; OR, odds ratio; P-adjust, p-value after Bonferroni correction; P-adjust<0.01, statistically significant.

LASSO regression identified 21 candidate drugs. These variables, together with baseline patient characteristics (age and sex), were included in a multivariable logistic regression model (Figure 4).

In the multivariable analysis, older age and female sex were associated with higher reporting odds of cholangitis. In addition, 21 drugs—including nivolumab, pembrolizumab, gemcitabine, paclitaxel, lenvatinib, atezolizumab, bevacizumab, and capecitabine—remained independently associated with increased reporting odds.

**FIGURE 5:**
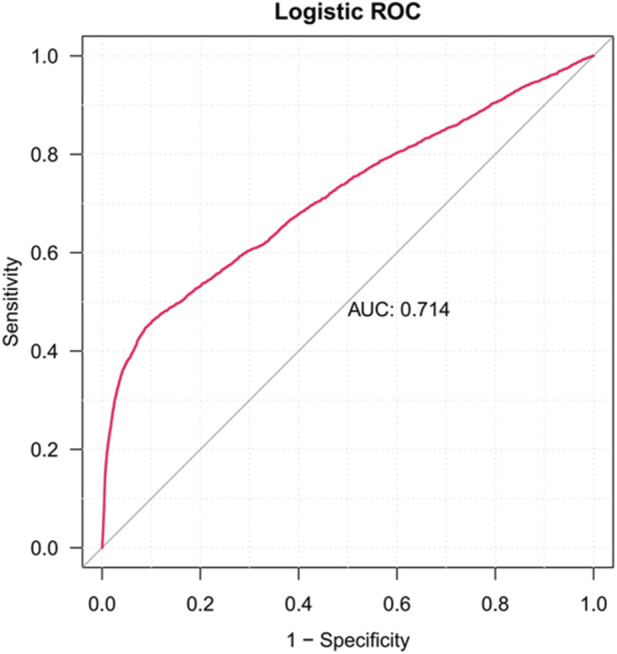
Receiver operating characteristic (ROC) curve of the factors associated with reporting of cholangitis. ROC, receiver operating characteristic; AUC, area under the curve.

The model demonstrated moderate discrimination, with an area under the receiver operating characteristic curve (AUC) of 0.714 (Figure 5).

### 3.4 Time-to-onset analysis

**FIGURE 6:**
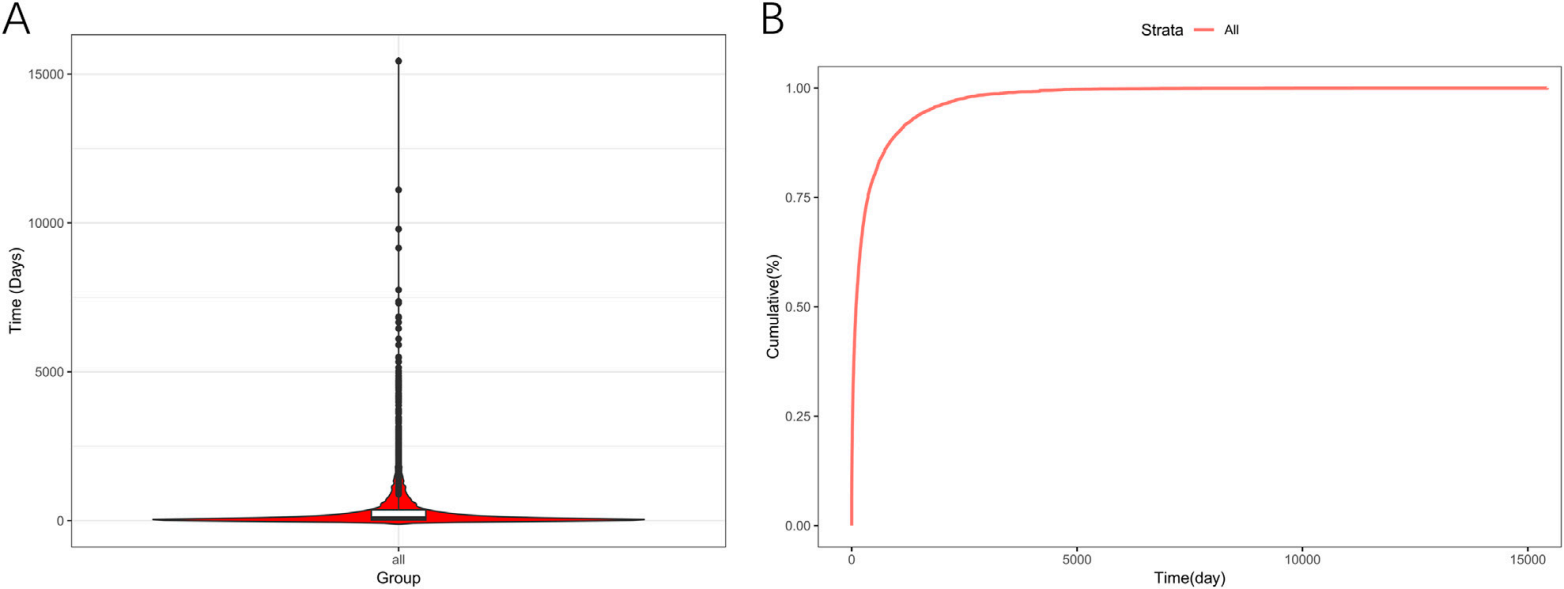
Time interval between drug intake and the onset of drug-related cholangitis. **(A)** Violin plot of time to drug-related cholangitis occurrence; **(B)** Cumulative incidence of drug-related cholangitis over 100 days.

Time-to-onset data were available for a subset of reports with complete therapy start and event dates. The distribution of time-to-onset is presented in Figure 6.

The median time to onset was 97 days (interquartile range [IQR], 7–102 days). A substantial proportion of cases occurred within the first 3 months following drug initiation.

The selected drugs were categorized into the following classes: antineoplastic agents (10/21), immunomodulators (5/21), antidiabetic drugs (3/21), antiviral agents (1/21), glucocorticoids (1/21), antibacterial agents (1/21), and systemic hormonal preparations excluding sex hormones (1/21) (Supplementary Table S3).

### 3.5 Sensitivity analysis

Several sensitivity analyses were performed to evaluate the robustness and consistency of the detected pharmacovigilance signals.

When the analysis was restricted to reports submitted by healthcare professionals, the majority of major disproportionality signals remained detectable, particularly among immune checkpoint inhibitors, tyrosine kinase inhibitors, antimetabolites, and anti-angiogenic agents. Although signal magnitudes were modestly attenuated in some cases, the overall direction and ranking of the principal signals were largely preserved.

Similarly, analyses limited to reports with complete demographic information yielded comparable results, with nivolumab, pembrolizumab, lenvatinib, gemcitabine, bevacizumab, and drospirenone-containing agents remaining among the most consistently reported drugs associated with cholangitis.

Application of more stringent signal detection thresholds (ROR ≥3 and Bonferroni-adjusted p < 0.01) reduced the total number of statistically significant signals but retained several clinically plausible agents previously supported by published case reports and mechanistic evidence.

Exploratory analyses excluding drugs primarily indicated for hepatobiliary malignancies or biliary tract disorders demonstrated partial attenuation of several signals; however, most key immune-mediated and targeted therapy-related signals remained statistically significant, suggesting that the primary findings were not solely attributable to indication-related confounding.

Overall, these sensitivity analyses supported the relative stability and internal consistency of the principal pharmacovigilance signals identified in this study.

### 3.6 Subgroup robustness analyses

Subgroup analyses were conducted to evaluate the consistency of reporting patterns across demographic categories.

Stratified analyses by sex demonstrated that several immune checkpoint inhibitors and targeted anticancer agents showed comparable disproportionality patterns in both male and female patients, although certain hormonal agents demonstrated stronger reporting signals among female patients, consistent with their predominant clinical utilization.

Age-stratified analyses revealed that older adults generally exhibited higher reporting odds for cholangitis across multiple drug classes, particularly among antineoplastic and immunomodulatory therapies. In contrast, several antimicrobial- and hormone-related signals were relatively more prominent in younger and middle-aged populations.

Additional exploratory analyses stratified by reporter type demonstrated that signals derived from healthcare professional reports were generally directionally consistent with those observed in the overall dataset, although some lower-frequency signals were attenuated after restriction to medically confirmed reports.

Importantly, no major qualitative reversals of the principal pharmacovigilance signals were observed across subgroup analyses, supporting the overall robustness and reproducibility of the primary findings.

### 3.7 Case-level clinical characterization

To improve the clinical interpretability of the identified pharmacovigilance signals, representative case-level characteristics of cholangitis reports were further evaluated.

Among the cholangitis-related reports, hospitalization was the most frequently documented serious outcome, whereas a smaller proportion of cases involved life-threatening events or death. The majority of reports occurred in older adults, with a predominance of female patients in several drug classes, particularly hormonal agents and immune-mediated therapies.

Time-to-onset analysis demonstrated that many reports occurred relatively early after treatment initiation, although substantial variability was observed across drug classes. Immune checkpoint inhibitors and targeted anticancer therapies frequently demonstrated delayed or progressive onset patterns, whereas antimicrobial- and hormone-associated reports tended to occur earlier.

Concomitant use of potentially hepatotoxic medications was frequently observed, particularly among oncology patients receiving combination chemotherapy, immunotherapy, or targeted treatment regimens. In addition, several reports described underlying malignancy, biliary obstruction, liver metastasis, or prior hepatobiliary disease, highlighting the potential contribution of disease-related confounding and clinical complexity.

Where available, positive rechallenge information suggested partial or complete clinical improvement following drug discontinuation in a subset of reports. Rechallenge information was infrequently reported but was positive in several isolated cases involving immune checkpoint inhibitors and targeted therapies, providing limited supportive evidence for potential drug-event relationships.

Overall, the case-level characteristics demonstrated clinically plausible temporal patterns and outcomes but also emphasized the substantial heterogeneity and confounding inherent to spontaneous reporting data.

## 4 Discussion

### 4.1 Overview of key findings and clinical significance

The mechanisms underlying drug-related cholangitis are likely multifactorial and remain incompletely understood. Several non-exclusive pathways may contribute, including immune-mediated biliary injury, direct epithelial toxicity, microvascular ischemia, and alterations in bile composition or flow. These processes may converge to promote bile stasis, biliary obstruction, and subsequent inflammation (Cozma et al., 2024).

The diversity of implicated drug classes observed in this study suggests that drug-related cholangitis represents a shared clinical outcome arising from heterogeneous pharmacological mechanisms rather than a single disease entity.

### 4.2 Demographic disproportionality signals: age and sex

The FAERS database represents one of the largest global pharmacovigilance systems and is widely used to investigate suspected drug-related adverse events, including cholangitis. In this global pharmacovigilance analysis of 17,083 reports, the median age of patients with drug-related cholangitis was 59 years (interquartile range [IQR], 42–70), with the majority of cases occurring in individuals aged >40 years, consistent with findings from previous pharmacovigilance studies (Cozma et al., 2024).

The increased incidence of gallstone disease after the age of 40 may partly accounts for this observation, as it contributes to a higher risk of biliary obstruction and subsequent cholangitis (Shaffer, 2006). This age-related trend is further supported by mechanistic and epidemiological evidence. In addition, geographic and ethnic variations in gallstone prevalence may also influence the observed distribution of drug-related cholangitis, as the prevalence of gallstones differs substantially across populations (Ahmed, 2018).

Importantly, our multivariable analysis identified age as an independent risk factor for drug-related cholangitis, with risk increasing progressively with advancing age.

In our study, the number of reported cases of drug-related Cholangitis was higher among females than among males (8,451 vs.7,111). Consistently, multivariable analysis identified female sex as an independent risk factor for drug-related cholangitis. Previous studies have reported that the incidence of cholangitis is approximately twofold higher in females than in males across different age groups, with rates reaching up to 50% in women over 70 years of age (Cozma et al., 2024).

This sex-related disparity typically emerges after puberty and persists throughout adulthood, which may be attributed to the effects of sex hormones and sex-specific differences in hepatic cholesterol metabolism and biliary physiology. Estrogen has been shown to increase cholesterol saturation in bile and reduce bile acid synthesis, thereby promoting gallstone formation and biliary stasis, both of which are key predisposing factors for cholangitis (Maurer et al., 1989; Völzke et al., 2005; Cozma et al., 2024).

From a clinical perspective, these findings suggest that female patients, particularly those receiving high-risk medications, may require closer monitoring for early signs of biliary complications. The interaction between drug-induced alterations in bile composition and underlying sex-related susceptibility may further amplify the risk of cholangitis in female patients.

### 4.3 Drug classes associated with increased reporting odds

Our multivariable analysis identified several drug classes, including tyrosine kinase inhibitors (TKIs), immune checkpoint inhibitors (ICIs), cytotoxic chemotherapeutic agents, and immunosuppressants, as being associated with increased reporting odds of drug-related cholangitis. Emerging evidence from case reports and small cohort studies supports these associations, although the overall level of evidence remains limited.

Comparison with current SmPC information (Supplementary Table S4) demonstrated that several identified drugs already include hepatobiliary adverse reactions or gallbladder disorders in their prescribing information, whereas cholangitis itself is not explicitly listed for many signal-positive agents. These findings may therefore provide additional pharmacovigilance evidence for potential biliary safety concerns that warrant further evaluation.

### 4.4 Antineoplastic and immunosuppressive agents: immune and direct toxic mechanisms

The underlying mechanisms vary across drug classes but can be broadly categorized into several key pathogenic pathways. First, immune-mediated injury appears to play a central role, particularly in immune checkpoint inhibitor–related cholangitis, where disruption of immune homeostasis leads to T cell–mediated biliary inflammation (Björnsson and Andrade, 2022). Second, direct biliary epithelial toxicity and microvascular injury have been proposed for tyrosine kinase inhibitors and certain chemotherapeutic agents, potentially resulting in ischemic damage to the bile ducts (Barnett and Malafa, 2001). Third, certain agents may induce cholestasis or biliary obstruction through alterations in bile composition or epithelial function (Ml et al., 1998; Cirillo et al., 2005; Colvin et al., 2022; Cozma et al., 2024).

Collectively, these findings suggest that drug-related cholangitis is not driven by a single mechanism but rather represents a convergent pathological process involving immune dysregulation, direct epithelial injury, and impaired bile flow, ultimately leading to biliary inflammation and ductal damage.

In the present FAERS analysis, several immune checkpoint inhibitors (ICIs), including nivolumab, pembrolizumab, atezolizumab, and durvalumab, were identified as significant signals associated with cholangitis reporting. Increasing evidence suggests that ICIs may induce immune-mediated cholangitis and secondary sclerosing cholangitis, although the overall incidence remains low.

Previous studies have linked PD-1/PD-L1 inhibitors with biliary injury. Fouchard et al. (2019) reported three cases of cholangitis associated with nivolumab, durvalumab combined with tremelimumab, and pembrolizumab therapy, respectively. Clinical manifestations included abdominal pain, fever, cholestatic liver abnormalities, and biliary inflammation on imaging, with most patients showing improvement after corticosteroid and ursodeoxycholic acid treatment. Similarly, Kawakami et al. (2017) retrospectively analyzed 91 patients with metastatic or recurrent non-small cell lung cancer treated with nivolumab and identified three cases of immune-mediated cholangitis, further supporting a potential association between PD-1 inhibition and biliary injury.

A systematic review of 31 cases of PD-1 inhibitor–related sclerosing cholangitis demonstrated that nivolumab accounted for most reported cases, followed by pembrolizumab, avelumab, and durvalumab (Onoyama et al., 2020). The median onset time was 5.5 treatment cycles, and characteristic imaging findings included bile duct dilatation without obstruction, diffuse extrahepatic bile duct thickening, and multifocal intrahepatic biliary strictures. Notably, CD8^+^ T-cell infiltration surrounding the bile ducts was observed in a substantial proportion of pathological specimens, supporting an immune-mediated mechanism. However, corticosteroid responsiveness was generally poor, with an overall response rate of only 11.5%.

Several additional reports have provided further pathological evidence for ICI-related cholangiopathy. Zen et al. (2020) and Azami et al. (2022) demonstrated predominant CD8^+^ T-cell infiltration within the biliary epithelium and bile duct submucosa in patients with pembrolizumab- and atezolizumab-associated cholangitis, suggesting that PD-1/PD-L1 blockade may disrupt immune tolerance and promote T-cell–mediated cholangiocyte injury.

Consistent with these observations, our FAERS analysis identified disproportionality signals for several ICIs, including nivolumab, pembrolizumab, atezolizumab, and durvalumab. These findings further support the growing recognition of ICI-related cholangiopathy as an emerging immune-related adverse event. However, interpretation requires caution because patients receiving ICIs frequently have advanced malignancies, liver metastases, prior biliary interventions, or concurrent infections, all of which may independently increase the risk of cholangitis. Therefore, the observed associations should be considered hypothesis-generating and require confirmation in prospective clinical studies.

In the present FAERS analysis, several targeted anticancer agents, particularly lenvatinib and bevacizumab, were identified as significant signals associated with cholangitis reporting. Although the available evidence remains limited, increasing clinical observations suggest that these agents may contribute to biliary injury and secondary sclerosing cholangitis.

Previous studies have reported cholangitis and sclerosing cholangitis–like biliary abnormalities following treatment with lenvatinib and bevacizumab-containing regimens. Suoh et al. (2021) described a case of lenvatinib-associated cholangitis in which the patient developed jaundice and cholangitis secondary to bile stasis caused by treatment-related duodenal ulceration involving the papilla. Similarly, von (von Figura et al., 2009) reported secondary sclerosing cholangitis in a patient receiving paclitaxel combined with bevacizumab, while (Kusakabe et al., 2019) described chemotherapy-related sclerosing cholangitis characterized by biliary strictures and dilatation during treatment with paclitaxel and bevacizumab. In both reports, clinical improvement was observed following treatment discontinuation, supporting a potential drug-related contribution.

Several mechanisms have been proposed to explain targeted therapy–associated cholangitis. Antiangiogenic agents may induce ischemic injury to the biliary epithelium through disruption of the peribiliary vascular plexus, resulting in biliary inflammation, strictures, and cholestasis. In addition, treatment-related gastrointestinal mucosal injury and secondary biliary obstruction may further contribute to cholangitis development. These mechanisms are biologically plausible given the dependence of cholangiocytes on an intact microvascular blood supply.

Consistent with these observations, our FAERS analysis identified disproportionality signals for lenvatinib and bevacizumab, supporting a potential association between targeted therapies and cholangitis. However, interpretation requires caution because these agents are predominantly administered to patients with advanced malignancies who frequently have biliary obstruction, liver metastases, prior hepatobiliary interventions, and concomitant anticancer therapies, all of which may independently increase the risk of cholangitis. Therefore, the observed associations should be considered hypothesis-generating and require confirmation in prospective clinical studies.

In the present FAERS analysis, several cytotoxic chemotherapeutic agents, including gemcitabine, paclitaxel, oxaliplatin, and capecitabine, were identified as significant signals associated with cholangitis reporting. Although direct evidence remains limited, increasing clinical observations suggest that these agents may contribute to biliary injury and cholangitis development.

Previous studies have reported biliary complications associated with gemcitabine-based chemotherapy. (Morino et al., 2019) retrospectively analyzed patients receiving adjuvant chemotherapy for extrahepatic cholangiocarcinoma and observed cholangitis among the most common adverse events leading to treatment discontinuation. Similarly, paclitaxel-containing regimens have been implicated in chemotherapy-related sclerosing cholangitis. (Matsuo et al., 2015) reported a patient who developed multiple intrahepatic and extrahepatic biliary strictures during nab-paclitaxel therapy, while a recent pharmacovigilance study by (Qin et al., 2025) identified significant hepatobiliary safety signals for both paclitaxel and nab-paclitaxel, particularly involving cholangitis and biliary disorders.

Evidence linking capecitabine and oxaliplatin to cholangitis has also emerged. (Woodford et al., 2022) reported grade III cholangitis in patients with unresectable biliary tract cancer treated with capecitabine, whereas (Mir et al., 2012) observed multiple cases of cholangitis in patients receiving gemcitabine plus oxaliplatin for advanced cholangiocarcinoma. Collectively, these findings provide clinical support for the pharmacovigilance signals identified in the present study.

Several mechanisms have been proposed to explain chemotherapy-associated cholangitis. Direct cholangiocyte toxicity, endothelial injury, ischemic damage to the biliary epithelium, treatment-related immunosuppression, and secondary inflammatory responses may all contribute to biliary injury and subsequent cholangitis. In addition, cumulative toxicity and multidrug treatment regimens may further increase the risk of biliary complications in susceptible patients.

Consistent with these observations, our FAERS analysis identified disproportionality signals for several cytotoxic chemotherapeutic agents, including gemcitabine, paclitaxel, oxaliplatin, and capecitabine. However, interpretation requires caution because these drugs are frequently administered to patients with advanced malignancies who often have liver metastases, biliary obstruction, prior hepatobiliary interventions, and concomitant anticancer therapies. Therefore, the observed associations should be considered hypothesis-generating and require confirmation in future clinical and mechanistic studies.

In the present FAERS analysis, several immunosuppressive agents, including tacrolimus, everolimus, ciclosporin, and mycophenolate mofetil, were identified as significant signals associated with cholangitis reporting. Most available evidence supporting these associations has been derived from transplant-related cohorts and observational studies.

Previous studies have suggested a potential relationship between immunosuppressive therapy and biliary complications following transplantation. De Simone et al. (2009) reported cholangitis as a cause of treatment failure in a cohort of liver transplant recipients converted from calcineurin inhibitors to everolimus. Similarly, Akamatsu et al. (2021) conducted a multicenter retrospective study involving liver transplant recipients and identified cyclosporine use as an independent risk factor for recurrent sclerosing cholangitis. Further support was provided by a meta-analysis of 13 retrospective cohort studies comprising 5,077 patients, in which mycophenolate mofetil and cyclosporine were associated with an increased risk of recurrent primary sclerosing cholangitis after liver transplantation (Chen et al., 2020).

Case-based evidence has also linked immunosuppressive agents to cholangitis and other biliary complications. Simic et al. (2006) described a transplant recipient receiving long-term cyclosporine therapy who subsequently developed cholangitis and secondary biliary cirrhosis, suggesting that drug-induced cholestasis may contribute to biliary injury and gallstone formation.

Several mechanisms may explain the association between immunosuppressive therapy and cholangitis. Chronic cholestatic injury, impaired biliary epithelial repair, altered bile composition, and increased susceptibility to biliary infection resulting from immunosuppression may all contribute to the development of cholangitis. In transplant recipients, these effects may be further compounded by ischemia–reperfusion injury, biliary strictures, altered biliary anatomy, and repeated endoscopic or percutaneous interventions.

Consistent with these observations, our FAERS analysis identified disproportionality signals for several immunosuppressive agents, including tacrolimus, everolimus, ciclosporin, and mycophenolate mofetil. However, interpretation requires particular caution because transplant recipients represent a highly complex population with multiple pre-existing risk factors for cholangitis. Consequently, the observed associations should be interpreted as hypothesis-generating and require further validation in prospective studies specifically designed to disentangle drug-related effects from transplantation-related complications.

However, interpretation of these signals is particularly challenging because transplant recipients frequently have complex hepatobiliary anatomy, ischemia-reperfusion injury, biliary reconstruction, prior biliary strictures, and repeated instrumentation, all of which independently increase the baseline risk of cholangitis. Concomitant administration of multiple immunosuppressive agents further complicates causal attribution.

Therefore, although the present findings suggest a possible association between immunosuppressive therapy and cholangitis, substantial residual confounding remains likely.

Mycophenolate mofetil (MMF), an ester prodrug of mycophenolic acid, is a widely used immunosuppressive agent in transplantation and autoimmune diseases. In the present FAERS analysis, MMF demonstrated a significant signal associated with cholangitis reporting, although the available clinical evidence remains limited.

Previous studies have reported biliary complications in patients receiving MMF-containing immunosuppressive regimens. Notably, Talwalkar et al. (2005) evaluated 30 patients with primary sclerosing cholangitis treated with MMF and reported treatment discontinuation due to adverse events in several patients, including one case complicated by bacterial cholangitis and sepsis. Although this observation does not establish a direct causal relationship, it suggests a potential association between MMF exposure and biliary complications in susceptible individuals.

The mechanisms underlying MMF-associated cholangitis remain poorly understood. Potential explanations include altered immune regulation, increased susceptibility to opportunistic infection, cholestasis, and indirect biliary epithelial injury related to chronic immunosuppression. However, these mechanisms are difficult to disentangle from the underlying clinical conditions of affected patients.

Consistent with previous observations, our FAERS analysis identified a disproportionality signal for MMF-associated cholangitis. Nevertheless, interpretation requires considerable caution because patients receiving MMF frequently have complex hepatobiliary conditions, including prior transplantation, biliary reconstruction, ischemia–reperfusion injury, pre-existing biliary strictures, and concomitant use of multiple immunosuppressive agents. These factors substantially increase baseline cholangitis risk and complicate causal attribution within spontaneous reporting databases. Therefore, the observed association should be considered hypothesis-generating and warrants further validation through dedicated clinical and mechanistic studies.

### 4.5 Antidiabetic agents: effects on biliary physiology

In this study, antidiabetic agents, including glucagon-like peptide-1 receptor agonists (GLP-1RAs) and dipeptidyl peptidase-4 (DPP-4) inhibitors, were identified as potentially associated with increased reporting odds of drug-related cholangitis.

In the present FAERS analysis, several glucagon-like peptide-1 receptor agonists (GLP-1 RAs), including liraglutide and semaglutide, demonstrated significant signals associated with cholangitis reporting. Although direct evidence linking GLP-1 RAs to acute cholangitis remains limited, substantial clinical evidence supports an association between GLP-1 RA therapy and gallbladder or biliary disorders.

Previous studies have consistently reported an increased risk of gallbladder-related adverse events among GLP-1 RA users. In the LEADER trial (Nauck et al., 2019), liraglutide treatment was associated with a significantly increased risk of acute gallbladder or biliary disease (HR 1.60, 95% CI 1.23–2.09). Similarly, a real-world study based on the United Kingdom Clinical Practice Research Datalink demonstrated a higher risk of gallbladder and biliary diseases among GLP-1 RA users compared with patients receiving other antidiabetic therapies (adjusted HR 1.79, 95% CI 1.21–2.67) (Nauck et al., 2019). Furthermore, a meta-analysis of 76 randomized controlled trials reported that GLP-1 RA therapy significantly increased the risk of gallbladder or biliary diseases (RR 1.37, 95% CI 1.23–1.52), including cholelithiasis (He et al., 2022).

Although these studies did not specifically evaluate acute cholangitis, the increased incidence of gallstone disease provides a biologically plausible explanation for the cholangitis signals observed in our analysis. Several mechanisms have been proposed, including impaired gallbladder motility, delayed gallbladder emptying, and rapid treatment-associated weight loss, all of which may promote bile stasis and gallstone formation. Subsequent biliary obstruction may then predispose susceptible individuals to ascending biliary infection and acute cholangitis.

Consistent with these observations, our FAERS analysis identified disproportionality signals for multiple GLP-1 RAs. However, interpretation requires caution because patients receiving GLP-1 RAs frequently have obesity, diabetes, and metabolic syndrome, conditions that independently increase the risk of gallstone disease and biliary complications. In addition, FAERS lacks detailed information regarding weight change, imaging findings, and pre-existing biliary disorders, limiting causal inference. Therefore, the observed associations should be considered hypothesis-generating and warrant further investigation in prospective clinical studies.

In contrast, dipeptidyl peptidase-4 (DPP-4) inhibitors did not show consistent or strong signals associated with cholangitis reporting (He et al., 2022). This finding aligns with the existing literature, which remains inconclusive: while some studies have reported increased reporting odds of cholecystitis, others have not identified a significant association with gallstones or cholangitis. From a mechanistic perspective, DPP-4 inhibitors may theoretically influence biliary physiology through incretin-related pathways, potentially affecting gallbladder motility; however, such effects appear to be modest and less pronounced than those observed with GLP-1 receptor agonists (Gasbjerg et al., 2020; Rehfeld et al., 2018). These findings should nevertheless be interpreted with caution. Differences in treatment duration, patient characteristics, and study design across datasets, as well as the relatively low event rates, may contribute to inconsistent results. In addition, FAERS data are subject to reporting bias and lack detailed clinical information, which limits the ability to detect subtle risk signals. Clinically, the current evidence does not support a clear association between DPP-4 inhibitors and cholangitis, but continued vigilance and further studies are warranted to better clarify their biliary safety profile.

Taken together, the available evidence suggests that GLP-1 receptor agonists may indirectly predispose patients to cholangitis by promoting gallbladder hypomotility, bile stasis, rapid weight loss, and subsequent gallstone formation. The present FAERS findings are therefore biologically plausible and broadly consistent with previous clinical and epidemiological observations regarding biliary complications associated with incretin-based therapies.

Nevertheless, causality remains difficult to establish because obesity, diabetes, metabolic syndrome, and weight reduction themselves are important independent risk factors for gallstone disease and biliary complications. In addition, FAERS lacks detailed imaging, longitudinal metabolic, and hepatobiliary data necessary for definitive assessment.

### 4.6 Other agents: microbiome and biliary dynamics

In the present FAERS analysis, doxycycline demonstrated a disproportionality signal associated with cholangitis reporting. Existing evidence supporting this association remains limited but suggests a potential link between doxycycline exposure and PSC-like cholangiopathy.

Buness et al. (2022) conducted a retrospective study involving 62 patients exposed to doxycycline and identified six cases with a temporal association between doxycycline exposure and the subsequent development of primary sclerosing cholangitis (PSC) or PSC associated with inflammatory bowel disease. In addition, one patient without prior long-term doxycycline exposure developed initial PSC-related symptoms during treatment. Across these seven cases, the median interval from doxycycline exposure to symptom onset was 6.5 months, whereas the median interval to formal PSC diagnosis was 24 months, suggesting a possible delayed and progressive biliary injury pattern.

Although causality remains uncertain, several biologically plausible mechanisms may explain this association. One potential pathway involves antibiotic-induced disruption of the gut microbiome and the gut–liver axis (Damman et al., 2018). Doxycycline may alter intestinal microbial composition and bile acid metabolism, thereby contributing to immune dysregulation and chronic biliary inflammation, processes that have been implicated in PSC pathogenesis (Ali et al., 2016). In addition, microbiome alterations may influence cholangiocyte homeostasis and susceptibility to persistent biliary injury.

However, these findings should be interpreted cautiously. The currently available evidence is predominantly observational and based on relatively small patient cohorts, limiting definitive causal inference. Furthermore, FAERS lacks detailed longitudinal clinical information, including microbiome profiling, imaging findings, histopathological confirmation, and baseline hepatobiliary status. Underlying inflammatory bowel disease, chronic infection, or other conditions requiring prolonged antibiotic therapy may also contribute to confounding. Clinically, these findings raise the possibility of a potentially underrecognized microbiome-mediated mechanism of drug-related cholangitis and suggest that clinicians should remain attentive to cholestatic abnormalities or biliary symptoms in patients receiving prolonged or repeated doxycycline therapy, particularly those with pre-existing gastrointestinal or hepatobiliary risk factors.

In the present FAERS analysis, octreotide and ceftriaxone were identified as significant signals associated with cholangitis reporting. Existing evidence suggests that both agents may contribute to biliary complications through alterations in bile composition and biliary motility.

Previous studies have demonstrated an increased risk of gallstone formation and biliary complications during long-term octreotide therapy. In a longitudinal study of patients with acromegaly receiving octreotide for more than 2 years, gallstone formation was observed in a substantial proportion of patients, and one individual developed cholestatic jaundice secondary to common bile duct stones (Plöckinger et al., 1990). More recently, Han et al. (2021) reported a case of acute cholangitis occurring shortly after octreotide administration, further supporting a potential association between octreotide therapy and biliary adverse events.

Evidence linking ceftriaxone to biliary complications is also well established. Shaffer (Ea, 2001) reviewed the phenomenon of drug-induced biliary sludge and highlighted both ceftriaxone and octreotide as agents capable of promoting sludge formation. Ceftriaxone may precipitate with calcium in bile to form insoluble complexes, resulting in biliary sludge or pseudolithiasis, whereas octreotide reduces gallbladder contractility and bile secretion, thereby promoting bile stasis. These alterations may subsequently lead to biliary obstruction and secondary cholangitis.

Consistent with these observations, our FAERS analysis identified disproportionality signals for both octreotide and ceftriaxone. The biological plausibility of these findings is supported by the well-recognized relationship between bile stasis, gallstone formation, biliary obstruction, and cholangitis. However, interpretation requires caution because patients receiving octreotide often have underlying neuroendocrine or gastrointestinal disorders, whereas ceftriaxone is frequently administered in the setting of severe infection or critical illness. These conditions may independently increase the baseline risk of biliary complications. Furthermore, the absence of imaging, microbiological, and histopathological information in FAERS limits detailed causality assessment.

Clinically, these findings highlight the importance of considering drug-induced biliary sludge, pseudolithiasis, and bile stasis in patients who develop new-onset cholangitis or unexplained cholestatic abnormalities during treatment with octreotide or ceftriaxone. Further studies are needed to clarify the magnitude of risk and identify patients who may be particularly susceptible to these adverse events.

In the present FAERS analysis, the combination of drospirenone (3 mg) and ethinylestradiol (20 μg) demonstrated a disproportionality signal associated with cholangitis reporting. Although direct evidence linking this formulation to cholangitis remains limited, the observed association is biologically plausible and broadly consistent with previous evidence suggesting that hormonal contraceptives may influence lipid metabolism and biliary physiology (Pehlivanov and Mitkov, 2007).

Previous studies have shown that drospirenone/ethinylestradiol therapy may alter lipid profiles, including increases in high-density lipoprotein cholesterol (HDL-C), whereas oral contraceptives containing more androgenic progestins, such as levonorgestrel, may exert different metabolic effects (Christin-Maitre, 2013). Drospirenone, a synthetic progestin structurally related to endogenous progesterone, also possesses antimineralocorticoid and antiandrogenic properties that may contribute to distinct metabolic and hepatobiliary effects compared with other progestins (Machado et al., 2011).

From a mechanistic perspective, estrogen-containing contraceptives may increase cholesterol saturation in bile, impair bile acid homeostasis, and promote biliary sludge or gallstone formation, thereby predisposing susceptible individuals to biliary obstruction and secondary cholangitis (Cozma et al., 2024). The present findings therefore support the possibility that drug-induced alterations in bile composition and biliary dynamics may contribute to cholangitis development in a subset of patients receiving hormonal therapy.

Nevertheless, these observations should be interpreted cautiously. Current evidence remains largely indirect and mechanistic, and users of hormonal contraceptives may differ in baseline metabolic status, body weight, and other risk factors for gallstone disease that could confound the observed association. In addition, FAERS lacks detailed information regarding duration of hormonal exposure, imaging findings, lipid metabolism, and pre-existing hepatobiliary disease, limiting definitive causal assessment. Clinically, these findings suggest that clinicians should remain attentive to potential biliary complications in women receiving hormonal contraceptives, particularly those with additional risk factors for gallstone disease or cholestatic disorders.

### 4.7 Time-to-onset characteristics and clinical implications

In our study, the median time to onset of drug-related cholangitis was 97 days (interquartile range [IQR], 7–102 days), with approximately 75% of cases occurring within the first 102 days after drug initiation (Von Figura et al., 2009; Gelsomino et al., 2018; Antón Rodriguez et al., 2024). Consistent with our findings, previous studies on drug-related cholangitis reports have similarly reported that most adverse events occur within the first 3 months of treatment.

These observations suggest that the risk of drug-related cholangitis may be highest during the early phase of drug exposure. However, because time-to-onset analyses were restricted to reports containing complete and chronologically plausible treatment and event dates, selection bias cannot be excluded. Reports with missing or inconsistent date information were necessarily omitted and may differ systematically from those included in the analysis.

### 4.8 Study strengths and limitations

Importantly, the incorporation of case-level clinical characterization improved the interpretability of the detected pharmacovigilance signals by identifying recurring temporal patterns, serious outcomes, and clinically plausible dechallenge observations across several major drug classes. Nevertheless, detailed causality assessment at the individual case level remained limited by incomplete reporting and the inherent constraints of spontaneous reporting systems.

Several important limitations should be considered when interpreting the present findings. First, FAERS is a spontaneous reporting database subject to substantial reporting bias, underreporting, duplicate reporting, notoriety bias, and variable report quality. The voluntary nature of reporting may preferentially capture severe, unusual, or recently recognized adverse events, thereby influencing disproportionality estimates.

Second, disproportionality analyses identify statistical reporting imbalances rather than causal relationships. Consequently, the observed signals should be regarded as hypothesis-generating pharmacovigilance observations rather than evidence of confirmed drug-induced cholangitis. Because denominator data and drug exposure information are unavailable in FAERS, true incidence rates and absolute risks cannot be estimated.

Third, important clinical details are frequently incomplete or unavailable, including imaging findings, microbiological data, laboratory parameters, histopathological confirmation, comorbidities, prior biliary disease, and concomitant medications. This limitation makes it difficult to distinguish true drug-related cholangitis from alternative etiologies such as malignant biliary obstruction, gallstone disease, infection, liver metastasis, or treatment-related complications.

Fourth, the present study restricted analyses to reports in which the investigated drug was designated as the primary suspect (PS) medication. Although this strategy improves signal specificity and reduces noise from concomitant therapies, it may exclude potentially relevant co-administered drugs and overlook multi-drug interactions or additive toxic effects. This limitation may be particularly important in oncology populations, where combination chemotherapy, immunotherapy, and targeted therapies are frequently administered simultaneously.

Additionally, substantial confounding may exist in specific clinical populations. Many of the identified signal-positive drugs, including gemcitabine, paclitaxel, bevacizumab, lenvatinib, and immune checkpoint inhibitors, are primarily administered to patients with advanced malignancies. These patients frequently have liver metastases, biliary obstruction, prior hepatobiliary interventions, concurrent infections, and exposure to multiple concomitant therapies, all of which may independently increase the risk of cholangitis. Therefore, confounding by indication and disease severity cannot be completely excluded.

Similarly, interpretation of signals involving immunosuppressive agents is complicated by the unique clinical characteristics of transplant recipients. Factors such as ischemia–reperfusion injury, altered biliary anatomy, biliary reconstruction, pre-existing strictures, repeated endoscopic instrumentation, and concomitant immunosuppressive regimens may independently contribute to cholangitis risk and complicate attribution to individual medications.

Furthermore, missing demographic information represents an additional limitation. Approximately one-quarter of reports lacked age information and more than half lacked body-weight data. Although missing values were retained in descriptive analyses whenever possible, exclusion from multivariable analyses may have introduced selection bias.

Finally, FAERS does not provide standardized confirmation of cholangitis through imaging, microbiological testing, endoscopic evaluation, or histopathological examination. The predictive model demonstrated moderate discriminative performance (AUC = 0.714), suggesting that additional unmeasured clinical factors not captured within FAERS likely contribute to the occurrence of drug-associated cholangitis. Consequently, the model should be viewed as exploratory rather than definitive. Consequently, diagnostic heterogeneity and potential case misclassification cannot be excluded.

## 5 Conclusion

This integrated pharmacovigilance assessment identified several drug classes with disproportionality signals suggestive of potential associations with cholangitis reporting in the FAERS database. The observed signals were supported to varying degrees by published clinical observations and mechanistic hypotheses; however, they should not be interpreted as evidence of causality. Rather, these findings represent pharmacovigilance signals that may help prioritize future clinical, epidemiological, and mechanistic investigations.

However, given the inherent limitations of spontaneous reporting systems, these findings should be interpreted as hypothesis-generating rather than confirmatory. Further validation through case-level analyses, pharmacoepidemiologic studies, and mechanistic investigations is required to better clarify causality and clinical relevance.

## Data Availability

The original contributions presented in the study are included in the article/Supplementary Material, further inquiries can be directed to the corresponding author.
