# Supplementary material for: Integrated pharmacovigilance assessment of drug-associated cholangitis: signal detection, clinical characterization, and mechanistic insights
Source: Front Pharmacol. 2026 Jul 30;17:1891926. doi: 10.3389/fphar.2026.1891926 (PMC13467760; doi:10.3389/fphar.2026.1891926)
Supplement: Supplementary file 1 [file Table1.docx]

Supplementary Table S1. Two-by-two contingency table for disproportionality analyses.

|  | Target adverse events | All other adverse events |
| --- | --- | --- |
| Target drug | a | b |
| All other drugs | c | d |
