# Supplementary material for: Integrated pharmacovigilance assessment of drug-associated cholangitis: signal detection, clinical characterization, and mechanistic insights
Source: Front Pharmacol. 2026 Jul 30;17:1891926. doi: 10.3389/fphar.2026.1891926 (PMC13467760; doi:10.3389/fphar.2026.1891926)
Supplement: Supplementary file 2 [file Table2.docx]

Supplementary Table S2. Cholangitis-related drugs reported in FAERS.

| **Drug Category** | **Drug** | **Number of Cases** | **ROR** | **95%CI** | **p-value** | **p-adjust** |
| --- | --- | --- | --- | --- | --- | --- |
| antineoplastic drugs | NIVOLUMAB | 573 | 10.25 | 9.43 - 11.14 | 0 | 0 |
|  | PEMBROLIZUMAB | 45 | 9.93 | 9.04 - 10.91 | 0 | 0 |
|  | GEMCITABINE | 309 | 13.08 | 11.68- 14.65 | 0 | 0 |
|  | PACLITAXEL | 269 | 8.33 | 7.38 - 9.4 | 0 | 0 |
|  | LENVATINIB | 201 | 9.92 | 8.63 - 11.41 | 0 | 0 |
|  | ATEZOLIZUMAB | 200 | 9.94 | 8.64 - 11.44 | 0 | 0 |
|  | BEVACIZUMAB | 194 | 3.09 | 2.68 - 3.56 | 3.93578E-60 | 3.98301E-57 |
|  | CAPECITABINE | 135 | 2.64 | 2.23 - 3.13 | 3.21958E-31 | 3.25822E-28 |
|  | DURVALUMAB | 107 | 11.4 | 9.42 - 13.81 | 4.4171E-217 | 4.4701E-214 |
|  | OXALIPLATIN | 106 | 3.62 | 2.99 - 4.39 | 9.76152E-45 | 9.87866E-42 |
|  | ERLOTINIB | 91 | 2.54 | 2.06 - 3.12 | 1.06719E-19 | 1.08E-16 |
|  | IRINOTECAN | 91 | 7.48 | 6.08 - 9.2 | 2.2704E-110 | 2.2977E-107 |
|  | IPILIMUMAB | 89 | 5.68 | 4.61 - 7 | 7.42774E-75 | 7.51687E-72 |
|  | FLUOROURACIL | 86 | 4.76 | 3.85 - 5.89 | 3.57234E-56 | 3.61521E-53 |
|  | SORAFENIB | 82 | 4.91 | 3.95 - 6.1 | 4.8274E-56 | 4.88533E-53 |
|  | REGORAFENIB | 59 | 7.02 | 5.43 - 9.07 | 2.84521E-66 | 2.87935E-63 |
|  | LETROZOLE | 51 | 2.79 | 2.12 - 3.67 | 5.93794E-14 | 6.0092E-11 |
|  | PANITUMUMAB | 48 | 5.31 | 3.99 - 7.05 | 3.65791E-37 | 3.7018E-34 |
|  | TIPIRACIL TRIFLURIDINE | 44 | 5.42 | 4.03 - 7.3 | 3.38976E-35 | 3.43044E-32 |
|  | PORFIMER | 42 | 166.04 | 120.04 - 229.66 | 9.91337E-76 | 1.00323E-72 |
|  | LAPATINIB | 31 | 2.91 | 2.05 - 4.15 | 1.22589E-09 | 1.2406E-06 |
|  | FOLINIC ACID | 27 | 6.85 | 4.69 - 10 | 2.71185E-14 | 2.7444E-11 |
|  | ENFORTUMAB VEDOTIN | 26 | 8.37 | 5.69 - 12.31 | 8.68259E-16 | 8.78678E-13 |
|  | CEMIPLIMAB | 16 | 8.66 | 5.3 - 14.17 | 1.6396E-10 | 1.65928E-07 |
|  | RAMUCIRUMAB | 14 | 4.07 | 2.41 - 6.88 | 1.57789E-05 | 0.01596824 |
|  | BICALUTAMIDE | 13 | 4.14 | 2.4 - 7.14 | 2.61464E-05 | 0.026460197 |
|  | FRUQUINTINIB | 11 | 6.23 | 3.44 - 11.26 | 2.69701E-06 | 0.002729376 |
|  | AVELUMAB | 8 | 4.15 | 2.07 - 8.31 | 0.000883138 | 0.893735932 |
|  | MITOMYCIN | 8 | 5.5 | 2.74 - 11.01 | 0.00014106 | 0.142753045 |
|  | VORINOSTAT | 6 | 4.84 | 2.17 - 10.8 | 0.001778673 | 1 |
|  | ZOLBETUXIMAB | 3 | 8.34 | 2.68 - 25.97 | 0.006015862 | 1 |
| antidiabetic drugs | SEMAGLUTIDE | 214 | 5.96 | 5.2 - 6.82 | 1.5469E-189 | 1.5655E-186 |
|  | SITAGLIPTIN | 162 | 6.66 | 5.7 - 7.77 | 1.5899E-167 | 1.609E-164 |
|  | LIRAGLUTIDE | 113 | 3.84 | 3.19 - 4.62 | 1.7848E-52 | 1.80622E-49 |
|  | METFORMIN SITAGLIPTIN | 53 | 7.15 | 5.45 - 9.37 | 5.13997E-61 | 5.20165E-58 |
|  | LINAGLIPTIN | 25 | 3.58 | 2.42 - 5.31 | 3.6833E-11 | 3.7275E-08 |
|  | GLIMEPIRIDE | 13 | 4.42 | 2.56 - 7.62 | 1.34053E-05 | 0.013566139 |
|  | INSULIN DEGLUDEC LIRAGLUTIDE | 7 | 8.98 | 4.27 - 18.9 | 1.80538E-05 | 0.018270416 |
|  | ALOGLIPTIN | 6 | 5.26 | 2.36 - 11.74 | 0.001173483 | 1 |
|  | NATEGLINIDE | 4 | 10.35 | 3.87 - 27.7 | 0.000698002 | 0.706378059 |
| immunomodulatory drugs | TACROLIMUS | 269 | 4.97 | 4.41 - 5.61 | 3.3276E-183 | 3.3675E-180 |
|  | VEDOLIZUMAB | 260 | 4.82 | 4.27 - 5.45 | 3.0229E-169 | 3.0592E-166 |
|  | MYCOPHENOLIC ACID | 200 | 3.67 | 3.19 - 4.22 | 9.07968E-85 | 9.18864E-82 |
|  | CICLOSPORIN | 122 | 2.69 | 2.25 - 3.22 | 2.14257E-29 | 2.16828E-26 |
|  | BASILIXIMAB | 11 | 7.46 | 4.12 - 13.49 | 4.85511E-07 | 0.000491337 |
|  | SATRALIZUMAB | 5 | 5.19 | 2.16 - 12.5 | 0.003158875 | 1 |
|  | EVEROLIMUS | 128 | 3.85 | 3.23 - 4.58 | 2.6548E-59 | 2.68666E-56 |
| Systemic Hormonal Preparations | OCTREOTIDE | 237 | 10.81 | 9.51 - 12.3 | 0 | 0 |
|  | LANREOTIDE | 94 | 12.63 | 10.3 - 15.49 | 8.0581E-215 | 8.1548E-212 |
|  | ETELCALCETIDE | 24 | 10.74 | 7.18 - 16.06 | 5.10286E-17 | 5.1641E-14 |
|  | PASIREOTIDE | 23 | 12.02 | 7.97 - 18.13 | 2.13174E-17 | 2.15733E-14 |
|  | PEGVISOMANT | 17 | 5.93 | 3.68 - 9.55 | 1.19279E-08 | 1.2071E-05 |
| antibacterial drugs | DOXYCYCLINE | 135 | 9.04 | 7.63 - 10.72 | 5.2488E-207 | 5.3118E-204 |
|  | CEFTRIAXONE | 90 | 9.65 | 7.84 - 11.88 | 8.621E-150 | 8.7245E-147 |
|  | AMOXICILLIN CLAVULANIC ACID | 45 | 3.23 | 2.41 - 4.33 | 3.19395E-16 | 3.23227E-13 |
|  | TERBINAFINE | 25 | 3.1 | 2.09 - 4.59 | 7.60454E-09 | 7.6958E-06 |
|  | CEFOPERAZONE SULBACTAM | 5 | 11.62 | 4.81 - 28.05 | 8.84028E-05 | 0.089463603 |
| antiatherosclerotic drugs | EZETIMIBE | 54 | 4.58 | 3.51 - 5.99 | 9.19842E-34 | 9.3088E-31 |
|  | FENOFIBRATE | 21 | 3.95 | 2.58 - 6.07 | 4.84667E-11 | 4.90483E-08 |
|  | EZETIMIBE SIMVASTATIN | 12 | 3.6 | 2.04 - 6.35 | 0.000188256 | 0.190514732 |
|  | FLUVASTATIN | 9 | 6.1 | 3.17 - 11.75 | 2.48315E-05 | 0.025129455 |
|  | EZETIMIBE | 54 | 4.58 | 3.51 - 5.99 | 9.19842E-34 | 9.3088E-31 |
| antiviral drugs | RIBAVIRIN | 52 | 3.21 | 2.44 - 4.22 | 2.2653E-18 | 2.29248E-15 |
|  | OMBITASVIR PARITAPREVIR RITONAVIR | 20 | 6.62 | 4.27 - 10.28 | 9.8546E-11 | 9.97286E-08 |
|  | ATAZANAVIR | 17 | 4.55 | 2.83 - 7.33 | 4.64757E-07 | 0.000470334 |
|  | DACLATASVIR | 12 | 4.25 | 2.41 - 7.49 | 4.14449E-05 | 0.041942238 |
|  | RITONAVIR TIPRANAVIR | 3 | 15.63 | 5 - 48.85 | 0.001047542 | 1 |
| Bile and Liver Therapy | URSODEOXYCHOLIC ACID | 79 | 51.17 | 40.82 - 64.14 | 1.296E-102 | 1.3116E-99 |
|  | ODEVIXIBAT | 3 | 18.46 | 5.9 - 57.79 | 0.00065275 | 0.66058251 |
|  | OBETICHOLIC ACID | 48 | 8.69 | 6.54 - 11.54 | 1.4255E-70 | 1.44261E-67 |
| Digestives, Including Enzymes | PANCREATIN | 90 | 7.51 | 6.1 - 9.25 | 8.6111E-110 | 8.7145E-107 |
| Enzyme Preparations | PANCRELIPASE | 11 | 5.35 | 2.96 - 9.67 | 1.09926E-05 | 0.011124477 |
| Anesthetics | KETAMINE | 46 | 26.68 | 19.91 - 35.76 | 4.54678E-48 | 4.60134E-45 |
| Drugs for Functional Gastrointestinal Disorders | MESALAZINE | 35 | 3.05 | 2.19 - 4.25 | 1.24731E-11 | 1.26228E-08 |
| Other Alimentary Tract and Metabolism Products | TEDUGLUTIDE | 58 | 5.69 | 4.4 - 7.37 | 2.75354E-49 | 2.78658E-46 |
| X-ray contrast media, non-water soluble | ETHIODIZED OIL | 27 | 32.64 | 22.25 - 47.86 | 3.69692E-31 | 3.74128E-28 |
| Drugs for Obstructive Airway Diseases | THEOPHYLLINE | 6 | 5.23 | 2.34 - 11.66 | 0.001215641 | 1 |
| Antigout Preparations | DAPRODUSTAT | 5 | 5.44 | 2.26 - 13.11 | 0.002585521 | 1 |
| Anti-inflammatory and antirheumatic products, non-steroids | ETODOLAC | 4 | 7 | 2.62 - 18.71 | 0.002859561 | 1 |
| Antipsoriatics | MIRIKIZUMAB | 4 | 10.21 | 3.81 - 27.32 | 0.000734162 | 0.742972201 |
| glucocorticoids | DROSPIRENONE ETHINYLESTRADIOL | 989 | 29.41 | 27.56 - 31.38 | 0 | 0 |
| Others | LANTHANUM CARBONATE | 11 | 5.01 | 2.77 - 9.07 | 1.97447E-05 | 0.019981687 |
|  | PLACEBO | 7 | 5.38 | 2.56 - 11.3 | 0.000412092 | 0.417036721 |
