# Supplementary material for: Integrated pharmacovigilance assessment of drug-associated cholangitis: signal detection, clinical characterization, and mechanistic insights
Source: Front Pharmacol. 2026 Jul 30;17:1891926. doi: 10.3389/fphar.2026.1891926 (PMC13467760; doi:10.3389/fphar.2026.1891926)
Supplement: Supplementary file 3 [file Table3.docx]

Supplementary Table S3. The categories of 36 drugs.

| **Drug Category** | **Drug** | **OR** | **95%CI** | **p-value** | **p-adjust** |
| --- | --- | --- | --- | --- | --- |
| antineoplastic drugs | NIVOLUMAB | 10.25 | 9.43 - 11.14 | <0.01 | <0.01 |
|  | PEMBROLIZUMAB | 9.93 | 9.04 - 10.91 | <0.01 | <0.01 |
|  | GEMCITABINE | 13.08 | 11.68- 14.65 | <0.01 | <0.01 |
|  | PACLITAXEL | 8.33 | 7.38 - 9.4 | <0.01 | <0.01 |
|  | LENVATINIB | 9.92 | 8.63 - 11.41 | <0.01 | <0.01 |
|  | ATEZOLIZUMAB | 9.94 | 8.64 - 11.44 | <0.01 | <0.01 |
|  | BEVACIZUMAB | 3.09 | 2.68 - 3.56 | <0.01 | <0.01 |
|  | CAPECITABINE | 2.64 | 2.23 - 3.13 | <0.01 | <0.01 |
|  | DURVALUMAB | 11.4 | 9.42 - 13.81 | <0.01 | <0.01 |
|  | OXALIPLATIN | 3.62 | 2.99 - 4.39 | <0.01 | <0.01 |
| immunomodulatory drugs | TACROLIMUS | 4.97 | 4.41 - 5.61 | <0.01 | <0.01 |
|  | VEDOLIZUMAB | 4.82 | 4.27 - 5.45 | <0.01 | <0.01 |
|  | MYCOPHENOLIC ACID | 3.67 | 3.19 - 4.22 | <0.01 | <0.01 |
|  | CICLOSPORIN | 2.69 | 2.25 - 3.22 | <0.01 | <0.01 |
|  | EVEROLIMUS | 3.85 | 3.23 - 4.58 | <0.01 | <0.01 |
| antidiabetic drugs | SEMAGLUTIDE | 5.96 | 5.2 - 6.82 | <0.01 | <0.01 |
|  | SITAGLIPTIN | 6.66 | 5.7 - 7.77 | <0.01 | <0.01 |
|  | LIRAGLUTIDE | 3.84 | 3.19 - 4.62 | <0.01 | <0.01 |
| glucocorticoids | DROSPIRENONE ETHINYLESTRADIOL | 29.41 | 27.56-31.38 | <0.01 | <0.01 |
| Systemic Hormonal Preparations | OCTREOTIDE | 29.41 | 27.56 - 31.38 | <0.01 | <0.01 |
| antibacterial drugs | DOXYCYCLINE | 9.04 | 7.63 - 10.72 | <0.01 | <0.01 |

CI, confid ence interval; OR, odds ratio; P-adjust, p-value after Bonferroni correction; P-adjust<0.01, statistically significant.
