# Supplementary material for: Integrated pharmacovigilance assessment of drug-associated cholangitis: signal detection, clinical characterization, and mechanistic insights
Source: Front Pharmacol. 2026 Jul 30;17:1891926. doi: 10.3389/fphar.2026.1891926 (PMC13467760; doi:10.3389/fphar.2026.1891926)
Supplement: Supplementary file 4 [file Table4.docx]

Supplementary Table S4. The SmPC of 21 drugs.

| **Drug Category** | **Drug** | **ROR** | **Cholangitis listed in SmPC** | **Hepatobiliary adverse events listed** |
| --- | --- | --- | --- | --- |
| antineoplastic drugs | NIVOLUMAB | 10.25 | No | Hepatitis |
|  | PEMBROLIZUMAB | 9.93 | Yes | Hepatitis, Clerosing cholangitis |
|  | GEMCITABINE | 13.08 | Yes | Elevated bilirubin, Elevated transaminase |
|  | PACLITAXEL | 8.33 | Yes | Elevated bilirubin, Elevated transaminase |
|  | LENVATINIB | 9.92 | Yes | Elevated bilirubin, Elevated transaminase |
|  | ATEZOLIZUMAB | 9.94 | No | Hepatitis, Elevated transaminase |
|  | BEVACIZUMAB | 3.09 | No | Gallbladder perforation |
|  | CAPECITABINE | 2.64 | Yes | Hyperbilirubinemia |
|  | DURVALUMAB | 11.4 | No | Hepatitis, Elevated transaminase |
|  | OXALIPLATIN | 3.62 | Yes | Elevated bilirubin |
| immunomodulatory drugs | TACROLIMUS | 4.97 | Yes | Hepatitis, Cholangitis |
|  | VEDOLIZUMAB | 4.82 | Yes | Elevated bilirubin, Elevated transaminase |
|  | MYCOPHENOLIC ACID | 3.67 | Yes | Elevated bilirubin |
|  | CICLOSPORIN | 2.69 | Yes | Bile stasis, Elevated bilirubin |
|  | EVEROLIMUS | 3.85 | No | liver damage |
| antidiabetic drugs | SEMAGLUTIDE | 5.96 | Yes | **Cholelithiasis** |
|  | SITAGLIPTIN | 6.66 | No | Elevated transaminase, Pancreatitis |
|  | LIRAGLUTIDE | 3.84 | Yes | **Cholelithiasis,** Cholecystitis |
| glucocorticoids | DROSPIRENONE ETHINYLESTRADIOL | 29.41 | Yes | **Cholelithiasis, C**holestasis |
| antibacterial drugs | DOXYCYCLINE | 9.04 | No | Hepatotoxicity, Pancreatitis |
| Systemic Hormonal | OCTREOTIDE | 10.81 | Yes | **Cholelithiasis,** Hyperbilirubinemia |

SmPC, Summary of Product Characteristics; ROR, Reporting Odds Ratio.
